# From Resection to Rehabilitation in One Day: Digital Workflow for Mandibular Reconstruction with Fibular Free Flap and Immediate Dental Rehabilitation Using CAD/CAM Guides at the Point of Care

**DOI:** 10.3390/cmtr19010015

**Published:** 2026-03-12

**Authors:** Matthias Ureel, Benjamin Denoiseux, Katrien Brijs, Pieter-Jan Boderé, Nicolas Dhooghe, Renaat Coopman

**Affiliations:** 1 Department of Oral & Craniomaxillofacial Surgery, Ghent University Hospital, C. Heymanslaan 10, 9000 Ghent, Belgium; benjamin.denoiseux@uzgent.be (B.D.); katrien.brijs@uzgent.be (K.B.); renaat.coopman@uzgent.be (R.C.); 2 Department of Dentistry, Ghent University Hospital, C. Heymanslaan 10, 9000 Ghent, Belgium; pieter-jan.bodere@uzgent.be; 3 Department of Plastic and Reconstructive Surgery, Ghent University Hospital, C. Heymanslaan 10, 9000 Ghent, Belgium; nicolas.dhooghe@uzgent.be

**Keywords:** three-dimensional (MESH), 3D printing, surgical cutting guide, free vascularised fibula graft, virtual surgical planning, mandibular reconstruction (MESH), immediate dental implant loading (MESH)

## Abstract

By using virtual surgical planning (VSP) and 3D printed guides, complex maxillofacial defects can be reconstructed with high accuracy and predictability. A fully digital workflow resulting in a modular all-in-one 3D printed guide system for fibula osteotomies, bone segment positioning, fully guided dental implant placement and dental prosthesis fixation for mandibular reconstruction was developed at Ghent University Hospital. A follicular ameloblastoma of the left mandible was resected in a 28-year-old male. The defect was reconstructed with a two-segment fibular free flap with immediate placement of three dental implants and immediate implant loading with a screw-retained bridge. A split thickness skin graft and Elemental PerioPlast were used as wound dressing. Comparison of the preoperative planning with the postoperative CT-scan showed a deviation immediately after surgery, which was no longer present at the 6-month follow-up. The patient achieved a stable occlusion and 44 mm mouth opening and reported high satisfaction. This case illustrates that fully digital, immediate mandibular reconstruction with simultaneous implant placement and prosthetic rehabilitation is feasible and accurate and enhances early functional recovery. Future improvements in intraoperative validation may further refine accuracy and reproducibility in complex oncologic reconstructions.

## 1. Introduction

Oral malignancies and aggressive benign lesions may infiltrate the maxilla and mandible, necessitating continuity resection and subsequent complex reconstruction. The fibular free flap (FFF) remains the gold standard for mandibular reconstruction due to its reliable vascular supply, adequate bone length, and favorable osteointegrative properties [1]. Over the past decades, the integration of virtual surgical planning (VSP) and patient-specific cutting and positioning guides has significantly enhanced precision, accuracy, and efficiency while reducing technical complexity [2].

Despite these improvements, a major limitation is still the delayed restoration of oral function, particularly dental occlusion and masticatory capacity. In response, ‘jaw-in-a-day’ (JIAD) protocols have emerged, aiming to achieve immediate oral rehabilitation and improved postoperative quality of life within a single surgical stage. Recent studies have confirmed that JIAD is no longer limited to benign cases but is a safe and feasible approach for malignant indications, provided there is meticulous planning for oncological margins [3,4].

A secondary challenge remains the reconstruction of the mandibular condyle. Restoring correct spatial orientation within the glenoid fossa is technically demanding [5]. The combination of microvascular mandibular reconstruction and alloplastic TMJ prosthesis has been described in the literature. To our knowledge, there has not been a report describing alloplastic TMJ reconstruction within the JIAD protocol [6,7,8].

Several techniques to perform the JIAD protocol such as the analog Rohner technique, stackable guides and 3D-printed patient specific osteosynthesis plates or a combination have been described [9,10,11,12,13,14,15,16,17]. Contemporary research highlights the superiority of fully digital workflows resulting in logical, robust and reproducible solutions for immediate functional rehabilitation [17].

In this report, we present a case of immediate mandibular reconstruction using a fibular free flap combined with simultaneous dental implant placement and prosthetic rehabilitation, based on a fully digital preoperative workflow at the point of care. This approach aims to integrate surgical precision with functional immediacy.

## 2. Materials and Methods

### 2.1. Patient Presentation and Preoperative Work-Up

**Figure 1:**
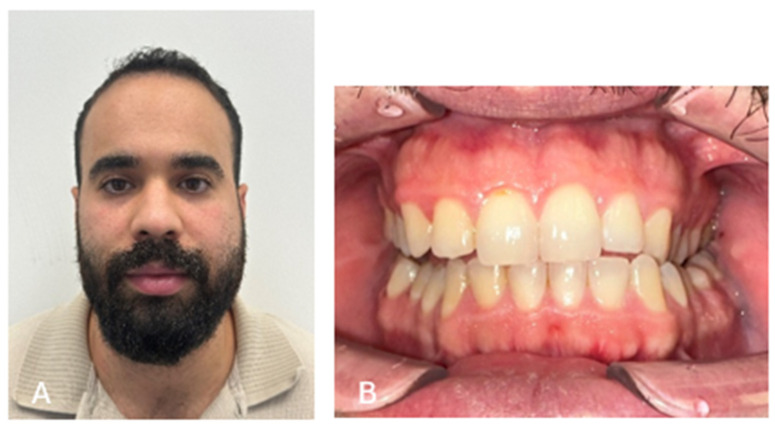
Initial clinical presentation with swelling of the left jaw. (**A**) Extra-oral swelling of the left jaw; (**B**) distortion of the occlusion due to the swelling.

**Figure 2:**
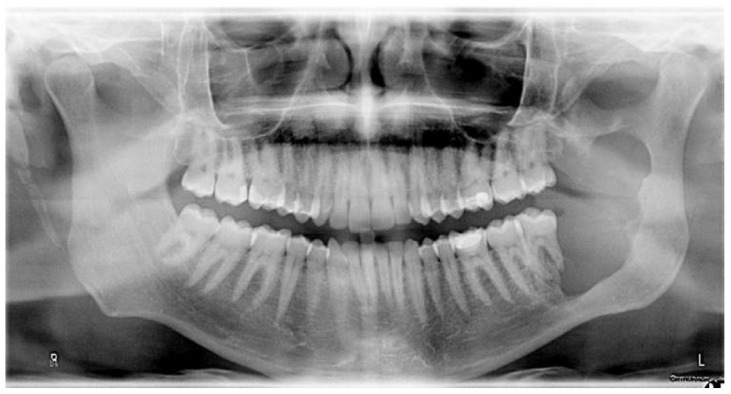
Preoperative orthopantomogram. A well-defined, scalloped radiolucent area is visible in the left mandible, extending towards the coronoid process. Teeth 37 and 38 are involved in the lesion.

A 28-year-old male presented with unilateral swelling and discomfort on the left side of the mandible (Figure 1). Clinical examination revealed a palpable buccal expansion in the left retromolar region. An orthopantomogram demonstrated a well-defined radiolucent lesion in the left mandibular body (Figure 2).

Radiographic evaluation with contrast-enhanced CT revealed a 6.6 cm × 2.5 cm expansile, lytic lesion located in the left mandibular body, extending posteriorly into the angle and ascending ramus. The lesion showed signs of cortical thinning, focal cortical perforation, and involvement of the roots of teeth 37 and 38. The mandibular canal was not clearly delineated within the lesion. No radiographic evidence of locoregional soft tissue invasion or thoracic metastases was observed.

An incisional biopsy confirmed the diagnosis of a follicular-type ameloblastoma. Genetic analysis for BRAF mutation was negative. A multidisciplinary surgical approach was planned involving both maxillofacial and reconstructive plastic surgery teams and consisted of a segmental mandibular resection, reconstruction with a fibular free flap and immediate prosthetic rehabilitation with dental implants and a screw-retained prosthetic bridge.

Prior to surgery, standardized clinical photographs were taken, and an occlusal registration in centric relation was obtained using a wax bite. Additionally, a facial scan (Metismile 3D Face Scanner, Shining 3D^®^, Hangzhou, China) and intraoral scans of both arches (Medit i500^®^, Medit, Seoul, Republic of Korea) were performed. A high-resolution thin-slice (0.6mm) maxillofacial CT scan (Somatom, Siemens, Munich, Germany) with the wax bite in situ, ensuring appropriate condylar seating and jaw relation, was taken. A CT angiography (dual-energy spiral CT, arterial phase of contrast injection) of the lower limbs was performed to assess vascular anatomy and donor site perfusion.

Using Materialise Enlight v6.0^®^ (Materialise NV, Leuven, Belgium), DICOM images were segmented and aligned with the intraoral scans to generate a virtual 3D twin of the patient’s maxillofacial and lower extremity structures.

### 2.2. Virtual Surgical Planning and 3D Printing

**Figure 3:**
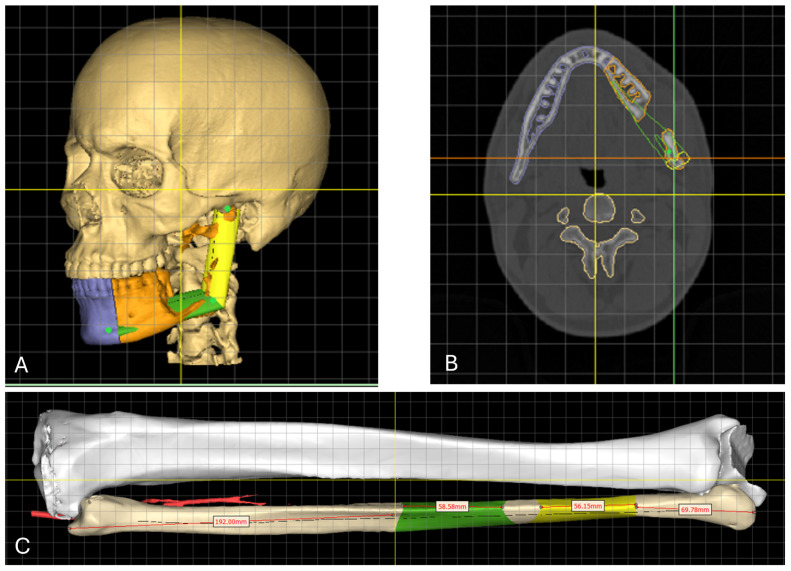
(**A**) Segmented 3D reconstruction of the skull (beige) and mandible, showing the native mandibular segment (purple) and the portion planned for resection (orange). The projected reconstruction with the free vascularized fibular graft is also visualized, with planned fibular segments shown in yellow and green. Reference lines in yellow.; (**B**) axial CT image with corresponding segmented overlays, color-coded to match the schematic in panel A. Reference lines in yellow, green and orange; (**C**) segmented CT angiography of the right lower leg, showing the tibia (white), native fibula and fibula segments (beige, yellow, and green). The vascular supply via the peroneal artery (a. fibularis) is indicated in red. The length of the fibula segments is visualized in white rectangles. Reference lines in yellow.

Given the extent of the lesion, the decision was made to place the resection margin distal to tooth 33 (Figure 3A–C), according to the Brown classification class Ic [18]. The left mandibular condyle was not preserved due to oncological considerations. Mandibular reconstruction was planned with a two-segment fibula graft from the right leg.

The alignment of the fibula segments was decided based on the anatomy of the patient and the position of the teeth (backward prosthetic planning). The intraoral scan and the dentition of the resected portion of the mandible (highlighted in orange in Figure 3A) served as anatomical reference points for segment positioning.

The virtual surgical plan (VSP) created in Materialise Enlight v6.0^®^ (Materialise NV, Leuven, Belgium) was exported to Materialise 3-Matic v18.0^®^ (Materialise NV, Leuven, Belgium) for the design and development of all surgical guides.

**Figure 4:**
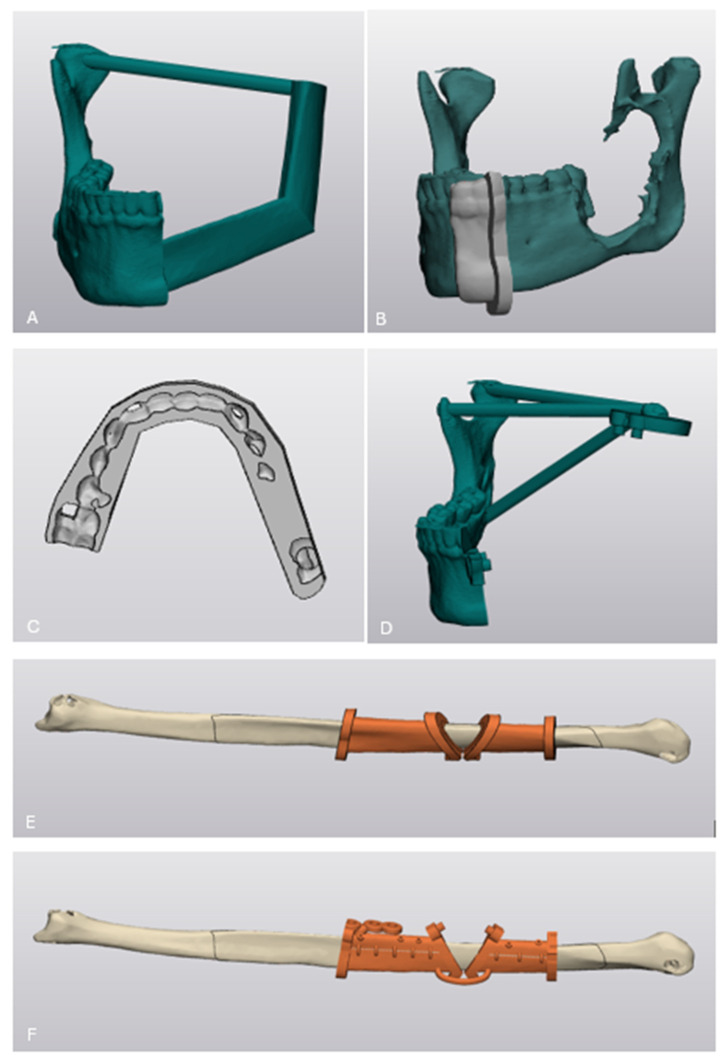
(**A**) Neomandible with an intercondylar reinforcement cylinder: (**B**) Gray cutting guide for mandibular osteotomy positioned on the neomandible (green); (**C**) interdental wafer to ensure correct positioning of reconstructed segments and screw-retained bridge; (**D**) mandibular repositioning guide; (**E**) basic version of fibula cutting guide (orange) positioned on the fibula bone (beige); (**F**) fibula cutting guide with modifications (orange) positioned on the fibula bone (beige).

The following guides were created:

1/The Neomandible: This anatomical model served as a template for pre-bending the 2.3 mm reconstruction plate prior to surgery. To increase structural rigidity during manipulation, an intercondyle cylindrical support (8 mm diameter) was incorporated (Figure 4A).

2/Cutting Guide for Mandibular Osteotomy: The cutting guide for oncologic resection of the left mandible consisted of a base plate (3 mm thickness) and a flange marking the osteotomy line distal to tooth 33 (Figure 4B).

3/Interdental Wafer: Using the Orthognatic module of Materialise Enlight v6.0^®^, a surgical wafer was designed based on the patient’s intraoral scans and preoperative occlusion. This wafer was intended to be used intraoperatively as a positioning guide to ensure accurate spatial orientation of the reconstructed segments (Figure 4C).

4/Mandibular Repositioning Guide: To ensure accurate positioning of the condylar segment, a dedicated repositioning guide was developed (Figure 4D). This guide was designed to interlock with the fibula cutting guide via matching pins and holes, allowing for precise seating of the condylar segment. Once the construct was stabilized, the pre-bent osteosynthesis plate could be positioned and fixated with KLS Martin 2.3 mm screws.

5/All-in-One Fibula Guide System: The fibula guide was designed in Materialise 3-Matic^®^ v18.0 using a standard workflow: the desired surface on the fibula was marked, a base plate with 3 mm thickness and an offset of 0.2 mm was created, and directional flanges to define the osteotomy lines were added. This guide served as the base for further adaptations (Figure 4E,F).

5a/The first adaptation of the fibula guide involved dividing the guide into superior and inferior parts using the ‘trim’ function. The inferior portion was designed to be removed intraoperatively after the osteotomy was performed, thereby creating space for placement of the pre-bent osteosynthesis plate. The superior and inferior parts were connected with 2 mm diameter cylinders using the ‘bridge’ module. Additional drill holes were incorporated into the superior parts to allow fixation of the guide onto the FFF graft using KLS Martin 2.0 mm screws. The guides of each fibula segment were connected with a larger 4 mm diameter cylinder on the inferior parts.

5b/Secondly, to prevent rotational displacement of the individual fibula segments, pins and holes were added to the flanges. The pins measured 3 mm in height and diameter, and the holes measured 3.1 mm in height and 3.1 mm in diameter. These components ensure that both fibula segments fit together in a mechanically interlocked and rotationally stable configuration. This pin-and-hole system was also added to the mandibular repositioning guide.

**Figure 5:**
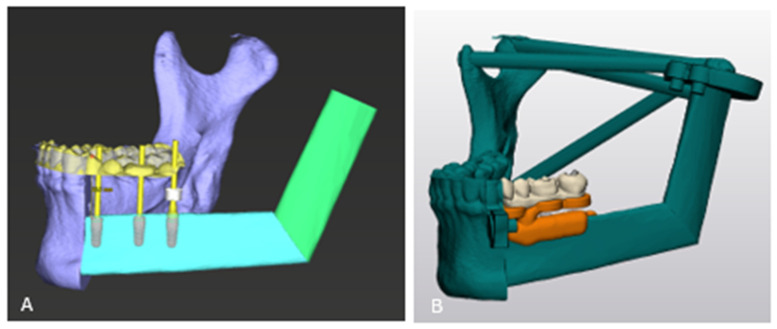
(**A**) Preoperative virtual surgical planning (VSP) for dental implant placement in the neomandible (purple, blue and green). Bredent^®^ implants (gray) are planned on teeth positions 34, 36 and 37. The intraoral scan (yellow) was used as a reference for positioning the dental implants in the fibula; (**B**) in Materialise 3-Matic^®^, the STL-file of the provisional bridge (beige) was imported and the fully guided implant drill guide (orange) was accurately transferred onto the fibula bone (green).

5c/The final adaptation involved the integration of fully guided implant sleeves (Figure 5A,B) for accurate dental implant placement. For this step, the coDiagnostiX^®^ software platform version 10.9 (Dental Wings GmbH, Berlin, Germany) was used. The relevant STL models were exported from Materialise Enlight^®^ v6.0 and imported into coDiagnostiX^®^. The intraoral scan of the mandible was used to determine the ideal implant positions. Three Bredent SKY Classic 4.0 × 10mm (Bredent medical, Senden, Germany) implants were virtually planned at positions 34, 36, and 37 (Figure 5A). A fully guided implant drill guide was designed and subsequently exported for further processing in Materialise 3-Matic^®^ v18.0. Using N-point alignment, the implant drill guide was accurately transferred onto the fibula cutting guide.

The provisional dental bridge was designed in Exocad^®^ (Exocad GmbH, Darmstadt, Germany) and based on the patient’s original dentition and anatomical references. Existing teeth served as the template for the occlusal morphology. The STL-file of the provisional bridge was imported into the virtual surgical plan (VSP), where it was digitally aligned with the pre-designed implant and fibular cutting guides. By subtracting the fibula guide from the provisional bridge, the bridge could be securely seated onto the fibula guide during surgery, enabling precise intraoperative fixation and immediate prosthetic loading (Figure 5B). This adaptation ensured precise spatial alignment and intraoperative control, allowing the correct occlusal vertical dimension (OVD) to be established during surgery.

All guides were 3D-printed in Ultrasint^®^ polyamide 11 (Forward AM Technologies, Heidelberg, Germany) with a Farsoon SS403P 3D printer (Farsoon Europe, Stuttgart, Germany). The provisional dental bridge was 3D-printed in polymer (Ultraprint Dental Denture UV, Heygears, Guangzhou, China—C&B Microfilled Hybrid, Nextdent, Soesterberg, The Netherlands) with a Heygears A3D 3D printer (Heygears, Guangzhou, China). The gingival component was colored manually to achieve an anatomically realistic appearance.

### 2.3. Surgery

#### 2.3.1. Tumor Resection

**Figure 6:**
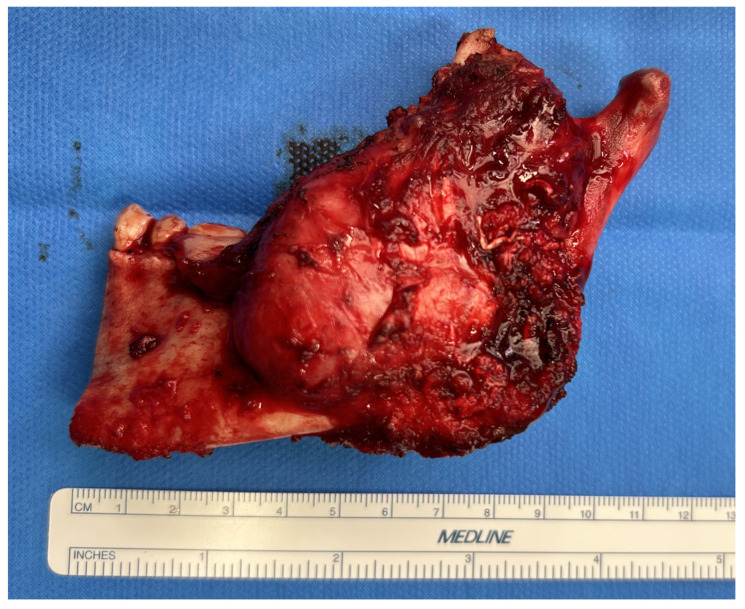
Surgical resection specimen of the ameloblastoma in the left mandible. The left condyle was completely resected.

A submandibular Risdon approach was used, and by ligating the facial vessels, the marginal facial nerve was preserved. Supraperiosteal dissection was preferred due to perforation of the mandibular cortex. A segmental mandibulectomy was performed starting from tooth 33 posteriorly, including the left condyle using the mandibular cutting guide. The mental nerve was transected and the lingual nerve, encased by the tumor, was sacrificed. The resection preserved the temporomandibular joint capsule. The resection specimen was submitted for histopathology (Figure 6).

#### 2.3.2. Fibular Free Flap Harvest

The osteomyocutaneous fibula free flap (FFF) was prepared from the right leg in a supraperiosteal plane with a mobile skin island.

**Figure 7:**
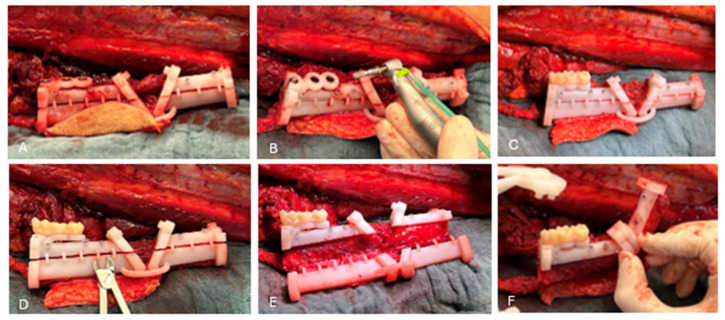
Osteotomy of the FFF and placement of dental implants according to the immediate loading protocol. (**A**) The fibular cutting guide was fixed to the right fibula using five KLS Martin 2.0 screws. The flanges were used to mark the osteotomy lines on the fibula with a piezotome. (**B**) The fully guided drilling protocol for the Bredent implants was executed using the incorporated drill sleeves. Three implants were placed. The implant drill guide was positioned at the correct height during the virtual surgical planning (VSP). (**C**) The provisional prosthesis was anchored to the three implants via 3mm unicone abutments and prosthetic cylinders using composite resin. Note that the prosthesis was seated directly onto the guide structure to ensure accurate positioning and vertical dimension. (**D**) The 2mm guide bridges were cut. (**E**) The lower part of the fibular cutting guide was removed. (**F**) The pin and hole connection system allowed accurate positioning of the two fibula flap segments. The fibula flap remained vascularized throughout the entire process.

The FFF all-in-one guide was then secured to the fibula using KLS Martin 2.0 screws (length: 9 mm). In accordance with the Bredent^®^ fully guided drilling protocol, three Bredent Classic SKY^®^ implants (4.0 × 10 mm, REF: kSKY4010) were placed into the fibula. Subsequently, four osteotomies were performed using soft tissue-friendly piezoelectric surgical instruments to avoid deperiostation of the fibula (Piezosurgery Flex, Mectron spa, Carasco, Italy) (Figure 7A,B). The provisional prosthetic bridge was accurately positioned on the all-in-one guide, restoring the dental anatomy of the patient and ensuring a correct vertical dimension (Figure 7B). Immediate loading was achieved with 3 mm straight unicone abutments (Ref: SKYUC003, Bredent medical, Senden, Germany) and plastic prosthetic cylinders (Ref: SKYUCPKK, Bredent medical, Senden, Germany). The prosthetic bridge was fixed using a light-cured flowable composite (G-ænial Universal Injectable, GC Europe, Leuven, Belgium) (Figure 7C).

**Figure 8:**
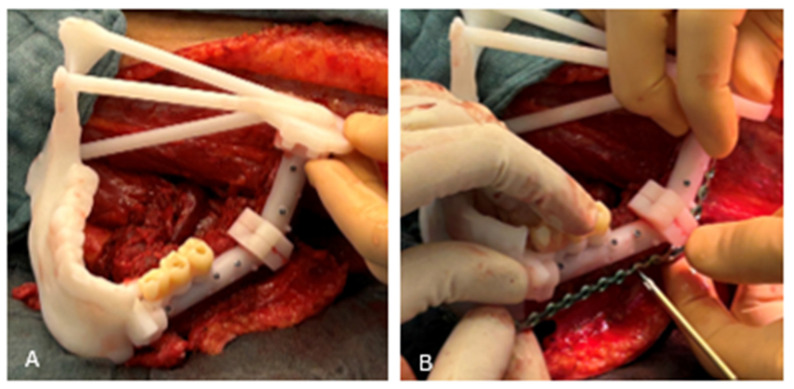
(**A**) The mandibular repositioning guide and fibular guide were connected via the pin-and-hole system. The fibula segments were now correctly positioned according to the VSP. (**B**) Removal of the lower portion of the fibular cutting guide created space for the placement of a reconstruction plate (KLS Martin 2.3^®^). This plate had been pre-bent on an anatomical model of the neomandible. Fixation was performed using KLS Martin 2.3^®^ screws. The FFF remained attached to its vascular pedicle during this step.

The 2 mm connecting cylinders of the guide were cut, and the inferior part of the fibula guide was removed, hereby mobilizing the fibula segments (Figure 7D–F). Both segments were positioned in the mandibular repositioning guide using pin-and-hole connections (Figure 8A).

The pre-bent KLS Martin 2.3mm reconstruction plate was secured to the fibula with locking and non-locking screws (Figure 8B). An additional 2.0 mm miniplate was placed anteriorly (KLS Martin 2.0^®^). The fibula construct was ready for transfer to the mandibula defect.

#### 2.3.3. Reconstruction

**Figure 9:**
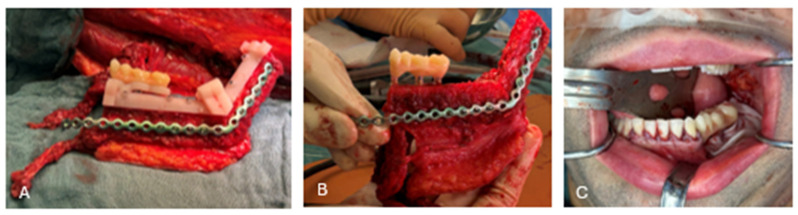
The completed fibular reconstruction. (**A**) After removing the mandibular repositioning guide, the fibula construct was visible. (**B**) The prosthetic restoration was removed from the implants to allow full removal of the fibula guide. Final adjustments were made to the dental bridge; the prosthesis was then polished and repositioned on the implants. (**C**) Intraoral image of the final situation. A split-thickness skin graft was placed around the dental implants and kept in place with an Elemental PerioPlast wound dressing placed below the screw-retained bridge.

The vascular pedicle was cut, the fibula guide was removed and the fibula construct, including the screw-retained prosthetic bridge, was transferred as a single unit into the oral cavity. The provisional prosthetic bridge served as an intraoperative guide to position the fibula construct in the occlusal plane to ensure proper alignment. Once optimal occlusion was confirmed, the reconstruction plate was fixated to the native mandible using KLS Martin 2.3 mm screws (Figure 9A,B). An additional KLS Martin 1.5 mm miniplate was placed for increased stability. Dental occlusion was verified using the interdental wafer.

Microvascular anastomoses were performed by the plastic surgery team, resulting in a total ischemia time of 115 min.

To optimize long-term implant maintenance, a split-thickness skin graft (STSG) was harvested from the right lower leg and placed intraorally over the implants to cover and protect the FFF periosteum. Fenestrations were created for implant emergence. Surfasoft and Elemental^®^ Perioplast (Withelemental, Ghent, Belgium) dressings were applied around the implants to stabilize the STSG and maintain a buccal vestibule [19]. The skin paddle of the FFF was sutured posteriorly to the implants in the retromolar area.

The remaining intraoral wounds were closed primarily, and the cervical and donor-site wounds were closed in a layered fashion (Figure 9C).

### 2.4. Postoperative Care Protocol and Flap Monitoring

The patient remained intubated overnight with planned extubation in the ICU the following day. Surgical drains were placed in the neck and fibular donor site. Prophylactic antibiotics (amoxicillin-clavulanic acid 1 g four times daily) were continued for seven days. The patient was kept nil per os (NPO) for the first five postoperative days. Oral hygiene began on postoperative day one using a sponge brush. Enoxaparin (Clexane^®^, Sanofi, Paris, France) was started eight hours postoperatively to prevent thromboembolism. Corticosteroids (methylprednisolone, Solumedrol^®^, Pfizer Inc., New York, NY, USA) were given to reduce edema.

Flap monitoring followed standard microvascular protocols. A MAP > 75 mmHg or systolic BP > 130 mmHg was maintained. The patient was positioned in a 30° Fowler position with the head in neutral midline orientation. Head rotation toward the flap side was avoided. Doppler ultrasound of the vascular pedicle was performed hourly for the first 24 h and then every two hours up to 72 h to detect early vascular compromise.

## 3. Results

**Figure 10:**
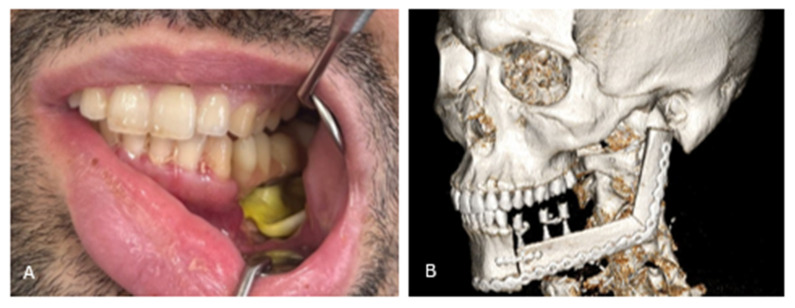
Clinical and radiological situation one week postoperatively. (**A**) Clinical picture with provisional screw-retained bridge. There was no dental occlusion posteriorly, most likely caused due to postoperative swelling. The Elemental^®^ wound dressing is visible under the provisional bridge and keeps the skin graft in place. (**B**) Aside from a small opening between the native mandible and the fibula graft, the postoperative CT scan shows a good alignment of the fibular graft with the native mandible. There is a diastasis between the distal end of the FFF and the glenoid fossa.

Immediate postoperative occlusion was deemed adequate, and as the postoperative edema subsided, the patient achieved a stable and functional occlusion. The Elemental^®^ Perioplast wound dressing was removed 12 days after surgery (Figure 10A).

The postoperative CT and orthopanthomogram demonstrated satisfactory alignment of the fibular graft with the native mandible (Figure 10B). A small gap between the fibula graft and native mandible was noticed. There was no evidence of loosening or mechanical failure. The dental implants were visualized in the correct position. On the postoperative CT scan, a diastasis was observed between the distal end of the FFF graft and the glenoid fossa.

**Figure 11:**
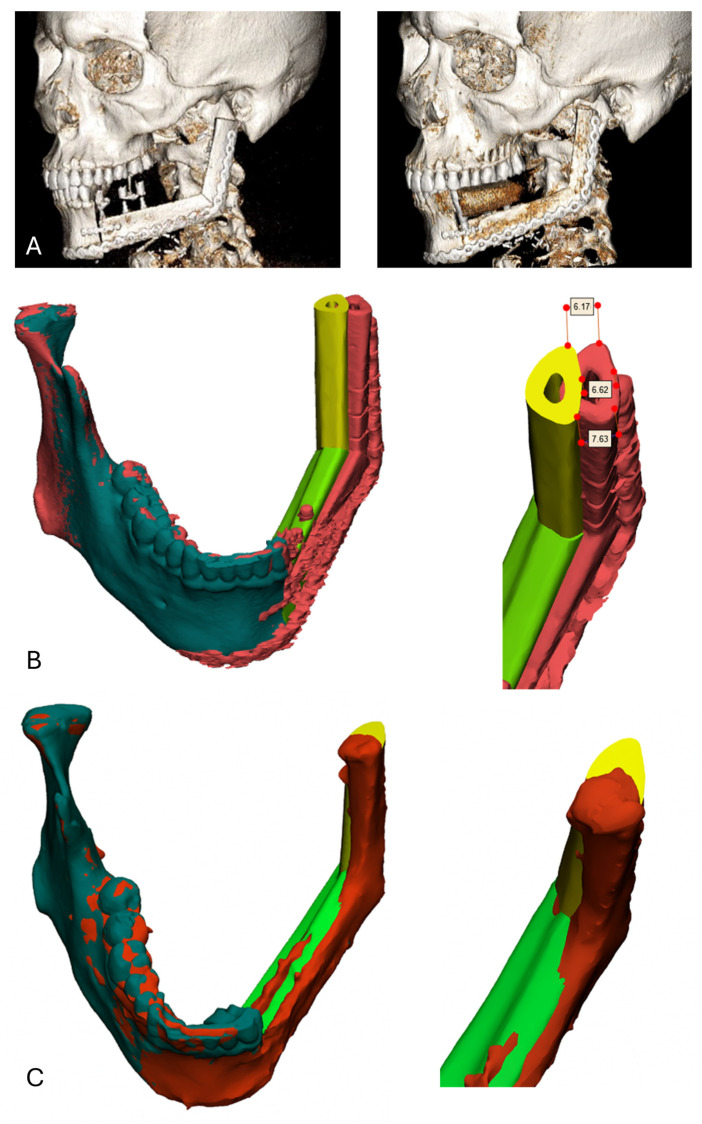
Postoperative analysis. Postoperative CT scan after one week and 6 months (**A**). (**B**) Comparison between the postoperative result (red) and the preoperative planning (mandible in green and yellow). A deviation of around 7 mm was observed due to a more lateral and caudal position of the neomandible compared to the preoperative planning. (**C**) Comparison after 6 months with a minimal deviation between postoperative result (red) and preoperative planning (green and yellow).

Using the ‘Part comparison’ module in Materialise 3-Matic^®^ v18.0 (Materialise NV, Leuven, Belgium), the preoperative virtual surgical plan (VSP) and the postoperative CT scan were analyzed to assess surgical accuracy. A maximal deviation of approximately 7 mm is seen at the free-ending fibula construct compared to the preoperative planning. The construct is positioned more laterally than planned (Figure 11A).

After six months, a new CT was performed to evaluate the position of the fibular fragments and osseous healing. A good osseous union was now observed, as well as beginning neocondylar formation due to the remodeling of the free end of the fibula [20]. The free end of the fibula had also shifted cranially towards the fossa as a result of the disappearance of oedema and medially probably due to occlusal guidance by the provisional dental bridge. A new comparison analysis showed a nearly perfect alignment between the preoperative planning and the postoperative result (Figure 11B).

Five months after surgery, the definitive prosthetic restoration was fabricated. High implant stability and healthy peri-implant soft tissues were observed. An intraoral digital impression was taken using Primescan^®^ (Dentsply Sirona, Mölndal, Sweden). A milled try-in was produced to evaluate occlusal relationships, vertical dimension, and esthetic parameters. Minor adjustments were performed as needed to optimize the functional and esthetic outcome.

**Figure 12:**
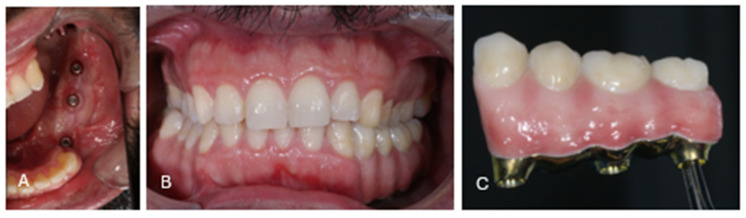
Clinical situation at 5 months postoperatively. (**A**) Excellent keratinization of the peri-implant mucosa is observed, facilitating adequate oral hygiene. (**B**) Clinical view showing the dental elements and prosthetic bridge in stable occlusion. (**C**) Close-up view of the definitive prosthetic bridge.

Given the patient’s young age and demonstrated ability to maintain proper oral hygiene, a screw-retained fixed prosthesis was chosen to maximize function, stability, and long-term tissue health. The final bridge consisted of a custom CAD/CAM-milled titanium framework onto which a full-contour zirconia bridge was bonded. The patient expressed high satisfaction with the efficiency and rapidity of the surgical recovery (Figure 12).

## 4. Discussion

This case illustrates the efficacy of a multidisciplinary approach in the management of head and neck reconstruction. The integration of virtual surgical planning (VSP), in-house 3D-printed surgical guides, and immediate dental rehabilitation enabled both functional and esthetic reconstruction. The fibular free flap (FFF) remains the preferred reconstructive option due to its osteointegrative capacity and adaptability to mandibular defects. Importantly, the placement of dental implants and immediate loading during the primary surgery significantly reduced overall rehabilitation time and improved the patient’s quality of life.

The evolution of 3D printing and VSP technologies has significantly expanded the possibilities of mandibular reconstruction. In contrast to earlier analog workflows such as the Rohner technique [10,21], which relied on plaster casts and manual planning, contemporary digital methods offer greater precision and reproducibility. The authors advocate for VSP protocols that adhere to four essential principles: accuracy, speed, reproducibility, and immediate rehabilitation.

- Accuracy: Surgeons must continuously strive for the highest level of accuracy in VSP execution. When surgical outcomes deviate from the plan, critical assessment of the VSP and refinement of clinical workflows are required.
- Speed: VSP should demonstrably reduce operative and ischemia time. Shorter ischemia times improve flap survival, and reduced operative durations limit anesthetic exposure and enhance postoperative recovery.
- Reproducibility: VSP must be embedded in a coherent digital workflow that is transferable to the surgical team, including residents and nursing staff. This enables consistent surgical outcomes and contributes to institutional learning.
- Immediate Rehabilitation: Particularly in oncological cases where adjuvant radiotherapy may hinder postoperative dental rehabilitation, immediate implant placement represents the future of jaw reconstruction [3,22,23]. While implants are often placed but not yet loaded, the proposed technique allows for controlled, reproducible, and accurate immediate functional rehabilitation—even in patients with limited prognosis—thus contributing to quality of life.

Certain considerations must be acknowledged regarding this approach. The guides must be designed with awareness of the surrounding soft tissues. The position of the vascular pedicle and potential perforators of the skin island or a muscle flap need to be taken into account. In cases necessitating a lot of soft tissue, it is sometimes difficult to position the fibula segments in the mandibular repositioning guide. Precautions not to rotate or compress the vascular pedicle should be taken. Also, during the preparation of the fibula construct, the pedicle should always be watched to avoid traction. Accurate positioning of the fibula all-in-one guide is important for adequate implant placement as the fibula is often thin and osseous perforations of the implants can occur. In Ghent University Hospital, all guides are designed by the surgeons themselves. While time-consuming, this approach fosters clinically driven innovation and enhances intraoperative adaptability by intimately understanding the limitations of the planning.

Another point of concern is the final positioning of the FF flap relative to the virtual plan. Although significant deviations were observed, they were not clinically relevant. This phenomenon has been reported by other authors as well [24,25,26]. While the condylar repositioning guide likely preserved correct spatial orientation, challenges persist during fixation to the native mandible, particularly in seating the distal segment accurately within the glenoid fossa. This final step often occurs after prolonged surgery, further complicated by soft tissue edema.

In this case, the provisional prosthetic bridge fractured under occlusal load a few days postoperatively. Two causes were identified: the use of plastic prosthetic components, which lacked mechanical strength, and reduced occlusal awareness due to postoperative sensory deficits. In consultation with the implant manufacturer, the plastic connectors were replaced with metallic components.

An interesting observation is the evolution of the spatial changes in the fibula construct over time. The postoperative CT scan after one week showed a significant lateral and caudal deviation of approximately 7 mm in the most distal part of the fibula construct compared to the preoperative planning. A small gap between the fibula and the native mandible was noticed at the fibula, suggesting suboptimal positioning of the fibula construct, although the occlusion was used as a guidance. Over time, six months later, the fibula construct positioned itself according to the virtual plan. The authors hypothesize that this change was induced by an optimalisation of the interdigitation of the prosthetic bridge with the native dentition in combination with intraoral muscle balances. The immediate placement of the prosthetic bridge served as a guide for correct osseous positioning; i.e., form follows function. This aspect warrants further investigation in future studies.

Future directions include refinements in surgical guide design and intraoperative validation workflows. A small window will be integrated into the implant drill guide to enable real-time visualization of implant placement and guide positioning on the fibula bone. The authors emphasize that achieving surgical outcomes significantly deviating from the original VSP undermines its purpose. Therefore, intraoperative imaging could serve as a valuable tool for immediate verification and adjustment of the plan. To enhance efficiency and precision, artificial intelligence may play a pivotal role in accelerating intraoperative image processing, currently limited by slow surface-based segmentation techniques.

## 5. Conclusions

Digital workflows enable accurate, single-stage mandibular reconstruction with simultaneous implant placement and immediate prosthetic rehabilitation (jaw-in-a-day). Dental occlusion can serve as a positioning tool for osseous reconstruction as form follows function. A functionally and esthetically stable result was achieved. Surgeon-driven VSP fosters innovation but requires experience and time investment. Future improvements should focus on intraoperative validation, potentially supported by artificial intelligence.

## Data Availability

The original contributions presented in this study are included in the article; further inquiries can be directed to the corresponding author.

## Abbreviations

The following abbreviations are used in this manuscript:

CAD/CAM	Computer-aided design/computer-aided manufacturing
FFF	Fibular free flap
ICU	Intensive care unit
NPO	Nil per os
OVD	Occlusal vertical dimension
QID	Quater in die
STSG	Split thickness skin graft
VSP	Virtual surgical planning

## References

[1] Hidalgo D.A. (1989). Fibula free flap: A new method of mandible reconstruction. Plast. Reconstr. Surg..

[2] Barr M.L., Haveles C.S., Rezzadeh K.S., Nolan I.T., Castro R., Lee J.C., Steinbacher D., Pfaff M.J. (2020). Virtual Surgical Planning for Mandibular Reconstruction with the Fibula Free Flap: A Systematic Review and Meta-analysis. Ann. Plast. Surg..

[3] Trac J., Ramchandani R., Dutta P., Sarkis L., Calvisi R., Villemure-Poliquin N., Davies J., Somogyi-Ganss E., Blanas N., Cuddy K. (2026). Jaw-in-a-day (JIAD) for malignant indications: A systematic review. Oral Oncol..

[4] Omole D., Khatib B., Patel A.A., Cheng A., Salama A., Brecht L.E., Hirsch D.L. (2023). Reconstructing the Mandible: Jaw-In-A-Day: Where We Were, Where We Are, and the Future. Atlas Oral. Maxillofac. Surg. Clin..

[5] Lee Z.H., Avraham T., Monaco C., Patel A.A., Hirsch D.L., Levine J.P. (2018). Optimizing Functional Outcomes in Mandibular Condyle Reconstruction With the Free Fibula Flap Using Computer-Aided Design and Manufacturing Technology. J. Oral Maxillofac. Surg..

[6] Chauvel-Picard J., Kreutzer K., Heiland M., Kreusch T., Ebker T., Beck-Broichsitter B. (2021). One stage microvascular mandible reconstruction by using scapula chimeric flap combined with computer-aided-design and computer-aided-manufacturing plate including bilateral alloplastic TMJ prosthesis: A case report. Microsurgery.

[7] Landes C., Korzinskas T., Dehner J.F., Santo G., Ghanaati S., Sader R. (2014). One-stage microvascular mandible reconstruction and alloplastic TMJ prosthesis. J. Cranio-Maxillofac. Surg..

[8] Pyne J.M., Davis C.M., Kelm R., Bussolaro C., Dobrovolsky W., Seikaly H. (2023). Advanced mandibular reconstruction with fibular free flap and alloplastic TMJ prosthesis with digital planning. J. Otolaryngol.—Head Neck Surg..

[9] Ureel M., Boderé P.J., Denoiseux B., Corthouts P., Coopman R. (2024). Mandibular Reconstruction with Osseous Free Flap and Immediate Prosthetic Rehabilitation (Jaw-in-a-Day): In-House Manufactured Innovative Modular Stackable Guide System. Bioengineering.

[10] Rohner D., Guijarro-Martínez R., Bucher P., Hammer B. (2013). Importance of patient-specific intraoperative guides in complex maxillofacial reconstruction. J. Cranio-Maxillofac. Surg..

[11] Williams F.C. (2023). History of the Jaw in a Day. Oral Maxillofac. Surg. Cases.

[12] Sukato D.C., Hammer D., Wang W., Shokri T., Williams F., Ducic Y. (2020). Experience With “Jaw in a Day” Technique. J. Craniofac. Surg..

[13] Schepers R.H., Raghoebar G.M., Vissink A., Lahoda L.U., Van der Meer W.J., Roodenburg J.L., Reintsema H., Witjes M.J. (2013). Fully 3-dimensional digitally planned reconstruction of a mandible with a free vascularized fibula and immediate placement of an implant-supported prosthetic construction. Head Neck.

[14] Khatib B., Cheng A., Sim F., Bray B., Patel A. (2020). Challenges With the Jaw in a Day Technique. J. Oral Maxillofac. Surg..

[15] Nayar S. (2019). Current concepts and novel techniques in the prosthodontic management of head and neck cancer patients. Br. Dent. J..

[16] Jeong Y.J., Dunn M., Manzie T., Howes D., Wykes J., Palme C.E., Leinkram D., Low T., Oberoi R., Aung Y.M. (2024). Jaw in a day surgery: Early experience with 19 patients at an Australian tertiary referral center. ANZ J. Surg..

[17] Van T.D., Khanh L.N., Dinh H.N., Phong M.H., Duc C.C. (2025). Fully Digital Workflow for Immediate Prosthetic Implant on Microsurgery Fibula Flap for Mandibular Reconstruction. Med. Arch..

[18] Brown J.S., Barry C., Ho M., Shaw R. (2016). Review A New classification for Mandibular Defects after Oncological Resection. Lancet Oncol..

[19] Van Der Kelen L., Ureel M., Denoiseux B., Boderé P.-J., Matthys C., Vermeersch H., Coopman R. (2025). Enhancing Implant Success in Mandibular Reconstruction: A Novel Approach Combining Implant-Retained Splint and Vestibuloplasty—A Case Series. J. Clin. Med..

[20] Lim M., Martinez I.A.V., Woods T., McIntyre B., Bayrakdar I.S., Kurt-Bayrakdar S., Jagtap R. (2025). Neocondylar Formation with Vascularized Fibular Free Flap: A Report of Three Rare Cases and Review of Literature. Surgeries.

[21] Rohner D., Bucher P., Kunz C., Hammer B., Schenk R.K., Prein J. (2002). Treatment of severe atrophy of the maxilla with the prefabricated free vascularized fibula flap. Clin. Oral Implant. Res..

[22] Wetzels J.W.G.H., Meijer G.J., Koole R., Adang E.M., Merkx M.A.W., Speksnijder C.M. (2017). Costs and clinical outcomes of implant placement during ablative surgery and postponed implant placement in curative oral oncology: A five-year retrospective cohort study. Clin. Oral Implant. Res..

[23] In’T Veld M., Schulten E.A.J.M., Leusink F.K.J. (2021). Immediate dental implant placement and restoration in the edentulous mandible in head and neck cancer patients: A systematic review and meta-analysis. Curr. Opin. Otolaryngol. Head Neck Surg..

[24] Coppen C., Verhoeven T., Snoeijink T., Weijs W., Verhulst A., van Rijssel J., Maal T., Dik E. (2025). Fibula free flap reconstruction: Improving the accuracy of virtual surgical planning using titanium inserts. Int. J. Oral Maxillofac. Surg..

[25] Blanc J., Fuchsmann C., Nistiriuc-Muntean V., Jacquenot P., Philouze P., Ceruse P. (2019). Evaluation of virtual surgical planning systems and customized devices in fibula free flap mandibular reconstruction. Eur. Arch. Oto-Rhino-Laryngol..

[26] Roser S.M., Ramachandra S., Blair H., Grist W., Carlson G.W., Christensen A.M., Weimer K.A., Steed M.B. (2010). The accuracy of virtual surgical planning in free fibula mandibular reconstruction: Comparison of planned and final results. J. Oral Maxillofac. Surg..

